# Glances at the Hospitals

**Published:** 1904-08-27

**Authors:** 


### GLANCES AT THE HOSPITALS.

### ROYAL EDINBURGH HOSPITAL FOR SICK CHILDREN.

The ordinary income amounted to £5,494, and the ordinary expenditure to £8,958, showing a deficiency of ordinary income of not less than £3,463, a rather alarming state of the finances; and it would seem that the ordinary income was lower than in the previous year and the number of patients higher. It should be noticed that legacies to the amount of £6,892 were received during the year and that special donations amounted to £1,645; so that things are not so bad as they appear at first sight. The hospital contained 81 patients on the first of January, and 1,597 were admitted during the year, making a total of 1,678 under treatment. Of these 1,393 were discharged, 191 died, and 94 remained in the hospital. The average daily number resident was 95; and the average stay in the wards was 20 days. The tables are very well arranged. The number of operations performed in the surgical theatre was 480; in the out-patients' department 920 minor operations were performed under anæsthetics, and 12,769 prescriptions were issued. Edinburgh being a great centre of medical education, it follows that the Sick Children's Hospital is made use of for clinical instruction, and last year 151 students attended the clinique.

### THE LEICESTER INFIRMARY.

The total income was £17,438, of which the workpeople contributed £4,984. The total ordinary expenditure was £15,604; but the total expenditure exceeded the total income by £810. The committee recognise the fact that the annual subscriptions must constitute the mainstay of the infirmary, and they add that "notwithstanding the great increase in the wealth and population of Leicester they are no larger than they were thirty years ago." Certainly this must be remedied if the infirmary is to retain its position. On January 1st 141 patients remained in the wards, and 2,174 were admitted during the year, making a total of 2,315. Of this number 1,828 were discharged cured or relieved, 79 were discharged not relieved, 122 for various reasons, 144 died, and 142 remained in the infirmary at the end of the year. In addition to the above there were treated in the children's hospital 582 patients, of whom 436 were discharged cured or relieved, 55 were discharged for various reasons, 47 died, and 44 remained under treatment. The average duration of each patient's stay was 22 days, and the daily average number of patients was 175. The average cost of each in-patient was £4 9s. 5d., and the average cost per week was £1 8s. 5d. So far the statistics are very clearly stated; but when we come to the medical and surgical tables we find that the information given is extremely meagre, but we believe that these statistics are printed in a separate report, in which case we hope to have an opportunity of referring to the subject in some future number.

